# A Bayesian Method to Incorporate Hundreds of Functional Characteristics with Association Evidence to Improve Variant Prioritization

**DOI:** 10.1371/journal.pone.0098122

**Published:** 2014-05-20

**Authors:** Sarah A. Gagliano, Michael R. Barnes, Michael E. Weale, Jo Knight

**Affiliations:** 1 Centre for Addiction and Mental Health, Toronto, Ontario, Canada; 2 Institute of Medical Science, University of Toronto, Toronto, Ontario, Canada; 3 William Harvey Research Institute, Barts and The London School of Medicine and Dentistry, Queen Mary University of London, London, United Kingdom; 4 Department of Medical & Molecular Genetics, King’s College London, Guy’s Hospital, London, United Kingdom; 5 Department of Psychiatry, University of Toronto, Toronto, Ontario, Canada; University of North Carolina, United States of America

## Abstract

The increasing quantity and quality of functional genomic information motivate the assessment and integration of these data with association data, including data originating from genome-wide association studies (GWAS). We used previously described GWAS signals (“hits”) to train a regularized logistic model in order to predict SNP causality on the basis of a large multivariate functional dataset. We show how this model can be used to derive Bayes factors for integrating functional and association data into a combined Bayesian analysis. Functional characteristics were obtained from the Encyclopedia of DNA Elements (ENCODE), from published expression quantitative trait loci (eQTL), and from other sources of genome-wide characteristics. We trained the model using all GWAS signals combined, and also using phenotype specific signals for autoimmune, brain-related, cancer, and cardiovascular disorders. The non-phenotype specific and the autoimmune GWAS signals gave the most reliable results. We found SNPs with higher probabilities of causality from functional characteristics showed an enrichment of more significant p-values compared to all GWAS SNPs in three large GWAS studies of complex traits. We investigated the ability of our Bayesian method to improve the identification of true causal signals in a psoriasis GWAS dataset and found that combining functional data with association data improves the ability to prioritise novel hits. We used the predictions from the penalized logistic regression model to calculate Bayes factors relating to functional characteristics and supply these online alongside resources to integrate these data with association data.

## Introduction

Genome-wide association studies (GWAS), which investigate the association between genetic variation and phenotypic traits, have identified many genes associated with human diseases [1]. However, despite considerable advances, much of the estimated heritability remains unaccounted for. Purcell et al. [2] showed that single nucleotide polymorphisms (SNPs) from GWAS with sub-threshold p-values account for a considerable proportion of the variance in independent samples suggesting that they are enriched for causal SNPs or their proxies. The issues of small sample size, low minor allele frequency, and lack of linkage disequilibrium (LD) between genotyped SNPs and the un-genotyped causal SNPs present challenges to detecting truly causal variants among near-significant genetic associations.

Emerging experimental data from various sources have suggested that the functional characteristics of specific genomic regions, such as histone modifications, DNase I hypersensitive sites, transcription factor binding sites, and expression quantitative trait loci (eQTL) among others, could offer biological explanations for many variants found to be associated with disease (for example: [3], [4], [5]). In September 2012, a series of publications from the ENcyclopedia of DNA Elements (ENCODE) Project Consortium, had the key message that much of the human genome, including non-coding and intergenic regions, overlaps with at least one functional element that may be active in certain cell types, under defined physiological conditions [6]. Furthermore, putative disease-causing variants show significant enrichment for multiple functional characteristics from the ENCODE Project [7]. For example, GWAS variants or variants with which they are in perfect LD are more frequently localized to DNase I hypersensitive sites than would be expected by chance [8].

Various tools are available that allow one to summarise the functional characteristics of variants in a given region. For instance, Boyle et al. developed RegulomeDB, a web-based interface that provides an easily interpretable score from an amalgamation of many functional characteristics derived from a variety of sources to annotate non-coding variants [9]. Other programs such as HaploReg [10] and SNPnexus [11] perform similar functions and account for LD. Although these programs provide facile access to summary information about the location of variants, they are only able to provide a relatively arbitrary and crude ranking of functional significance. The ranking scale used in RegulomeDB is based on the number of categories into which a variant falls with the highest scores given to those variants that fall into both an eQTL and a transcription factor binding site, regardless of cell type or specific transcription factor.

The central challenge in the interpretation of genetic associations lies in the processing and meaningful integration of a hugely diverse range of information. Having derived a score for a region containing a candidate variant, it has to be integrated with association evidence. We proposed the use of empirically derived weightings within a Bayesian framework [3]. More recently Schork et al. suggested the use of stratified False Discovery Rate (sFDR) and Darnell et al. proposed multi-thresholding in a manner that they say is equivalent to varying the significance threshold at each marker depending on the prior information [12], [13]. In order to implement these approaches one needs to define appropriate weights. For instance, Schork et al. [12] used an LD-weighted scoring algorithm, and Kindt et al. [14] recently published a multivariate logistic regression approach. However, neither of these approaches is easily scalable to the very large number of functional characteristics that are becoming available.

The primary objectives of this study are to describe an empirically justified method to identify which functional characteristics are best correlated with GWAS hit SNPs, to provide clues to the etiology of such traits, and to develop and implement a method to incorporate functional characteristics with statistical information in association studies. To achieve these objectives we use a machine learning approach, elastic net (a regularized logistic regression), to predict causality of a SNP based on information from 439 functional characteristics. We explore models based on all GWAS significant SNPs and also subsets of significant SNPs selected on the basis of phenotype and p-value. Functional characteristics are considered individually or in groups. We report a) the accuracy of the predictions to demonstrate the utility of the method and to investigate the behaviour of the different models, b) the frequency, correlation between and coefficients of the functional characteristics providing insight about their functional relevance to disease, c) a prediction score for each SNP, and d) details of how to combine this score with association statistics in a formal Bayesian framework.

We provide online scripts that can be employed so the method can be used by other researchers using additional functional characteristics (http://www.camh.ca/en/research/research_areas/genetics_and_epigenetics/Pages/Statistical-Genetics.aspx). For the best models we provide the probability of causality (the prediction score) for each SNP, the corresponding Bayes factor (BF_annot_) and scripts to combine BF_annot_ with GWAS association signals.

## Results

### Functional Enrichment in GWAS Hits

Frequencies of functional characteristics in GWAS hits compared to non-hits were compared using Fisher’s exact test. Our analyses indicate that GWAS hits are enriched for most functional characteristics compared to GWAS non-hits, except for splice sites and micro RNA (miRNA) targets, perhaps due to the very low frequency of these two classes of functional characteristics compared to the others (**Table 1** and **Table 2**).

**Table 1:** Summary statistics for the functional characteristics in the clumped non-phenotype specific analysis.

Description	Frequencyof annotationin GWAS hits	Frequencyof annotationin GWASnon-hits	p value(Fisher’s exact test)	OddsRatio	95% Confidenceinterval
splice	0.002	0.002	0.142	1.26	0.78–2.02
non-synonymous	0.022	0.007	2.38E-38	3.10	2.67–3.59
DNase Clusters	0.193	0.141	1.87E-39	1.46	1.38–1.54
GTEx eQTLs(all 7 experiments together)	0.020	0.007	1.69E-31	2.92	2.50–3.41
UK brain eQTLs	0.108	0.081	2.19E-18	1.37	1.28–1.47
UCSC Genes	0.422	0.357	7.36E-35	1.31	1.26–1.27
PhyloP*	0.217	0.172	6.56E-27	1.34	1.27–1.41
PhastCons*	0.243	0.202	3.63E-20	1.27	1.20–1.33
BroadHistone- H3k4Me1	0.637	0.566	2.20E-40	1.35	1.29–1.41
BroadHistone- H3k4Me3	0.509	0.434	1.63E-43	1.35	1.30–1.41
BroadHistone- H3k27ac	0.587	0.503	1.28E-53	1.48	1.34–1.46
Txn Factor ChIP(if annotation for any TF)	0.511	0.456	5.26E-24	1.25	1.10–1.14
miRNA	1.12E-4	7.00E-5	0.116	1.70	0.24–12.15
Gencode-Txn start sites	0.003	0.002	0.012	1.64	1.08–2.49

*As PhyloP and PhastCons conservation scores were left as quantitative measures, the frequencies reported for those characteristics represent the presence of a conservation score (ie. score>0).

**Table 2:** The mean score per SNP across all functional characteristics, classified by SNP type and functional variable type.

	Clumped	Separated
All SNPs	2.7	17.7
Hits	3.2	24.1
Non-hits	2.6	17.0

The histone modification data from the Broad Institute had the highest frequencies in GWAS hits, and the lowest p-values for enrichment. Many functional characteristics, most notably miRNA, were very infrequent, but the general picture was that their frequency in GWAS hits was greater than in GWAS non-hits.

**Figure 1:**
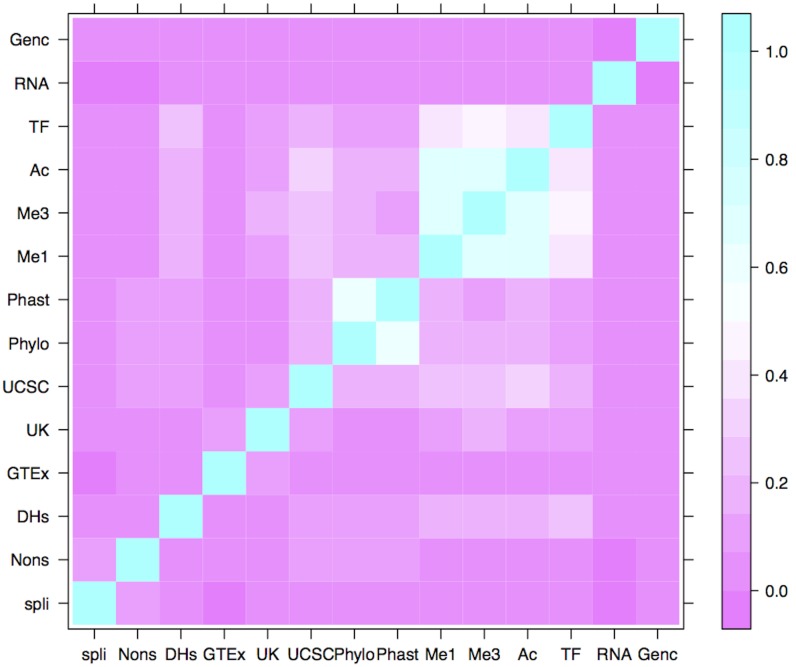
Heat map of correlations among the clumped functional characteristics. High correlations are seen between the two conservation measures PhyloP and PhastCons (represented as Phylo and Phast, respectively). Correlations are also seen among the histone modifications, H3k4Me1, H3k4Me3 and H3k27Ac (Me1, Me3 and Ac, respectively.) Transcription factor binding sites also show a correlation with the histone modifications. [spli = splice sites, Nons = nonsynonymous SNPs, DHs = DNase I hypersensitive sites, GTEx = cis-eQTL data from the GTEx Consortium, UK = cis-eQTL data from the UK Brain Consortium, Phylo = PhyloP conservation, Phast = PhastCons conservation, Me1 = H3K4Me1 histone modification, Me3 = H3K4Me3 histone modification, Ac = H3K27Ac histone modification, TF = transcription factor binding sites, RNA = micro RNA targets, Genc = transcription start sites from Gencode].

**Figure 2:**
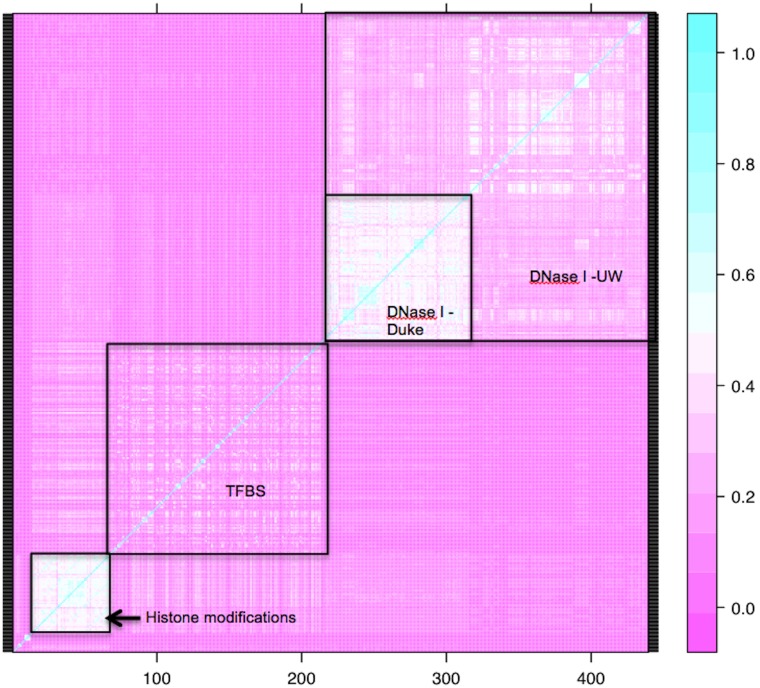
Heat map of correlations among the separated functional characteristics. A full list of the numbered characteristics is provided in Table S1. The white box in the bottom left corner corresponds to high correlation among the histone modifications. The less defined white area spanning from 72 to 219 on the x axis corresponds to correlation among the transcription factor binding sites, which also show some correlation with the histone modifications. The white box from 220 to 319 on the x axis corresponds to a high correlation among the different cell types for the DNase I hypersensitivity characteristic from Duke University. The less refined white box from around 320 and onwards on the x axis corresponds to the DNase I characteristics from the University of Washington. The plot also shows some correlation among the DNase I characteristics from both groups.

We examined the correlations among the various functional characteristics (**Figure 1** and **Figure 2**). The separated-variable analysis included measures of functional characteristics from different cell lines as individual factors, whereas the clumped-variable analysis grouped data from different cell lines for the same functional characteristic. The clumped analysis showed a strong correlation between the two conservation measures (PhyloP and PhastCons), as well as strong correlations among the three histone marks (H3k4Me1, H3k4Me3 and H3k27Ac), and to a lesser degree among the histone marks and transcription factor binding sites. The separated analysis revealed additional correlations among cell types investigated for the DNase I hypersensitive characteristics from Duke University, and to a lesser degree among the DNase I hypersensitive characteristics from the University of Washington, and between these two groups. These results highlight the issue of correlations among functional characteristics, many of which simply represent the same genomic feature, for example a promoter element measured by different technologies. One advantage of elastic net as a regularized logistic regression method is its ability to accommodate highly correlated variables.

### Predictive Accuracy of Functional Characteristics

We fitted predictive models for GWAS hit status via elastic net, using clumped and separated functional variable sets, using high-confidence (p<5×10^−8^) and low-confidence (p<10^−5^) GWAS hits, and using all GWAS hits (“non-phenotype specific”) as well as hits classified according broad phenotype areas. We primarily investigated predictive accuracy in a separate test set that was not involved in the fitting of the models.

For all of our fitted models, the area under the curve (AUC) of a receiver-operating characteristic (ROC) curve was similar in the test and training sets, suggesting that the models had not been over-fitted (**File S1, Part A**).

We found that the ROC curves for both the separated and clumped analyses had similar AUCs: for instance 0.58 in the test set for the non-phenotype specific clumped analysis and 0.59 in the test set for the separated analysis.

**Figure 3:**
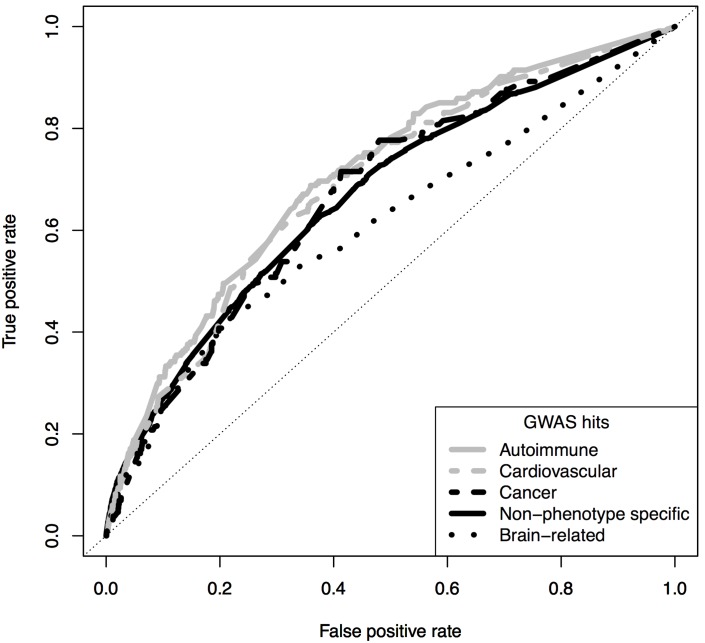
Receiver-operating characteristic (ROC) curves for analyses of clumped functional variables and high-confidence GWAS hits. ROC curves were obtained from a separate test set.

Two analyses emerged as most predictive based on integrating results from ROC curves, positive predictive values, and histograms of the probabilities of causality (the prediction scores). These were the analyses based on non-phenotype specific and the autoimmune GWAS analyses. Best results were obtained from analyses using high-confidence GWAS hits. Results for clumped and separated functional variables were very similar (**Table 3** and **Figure 3**).

**Table 3:** Areas under fitted ROC curves.

	Non-phenotype specific	Brain-related	Cancer	Cardiovascular	Autoimmune
N	3227 (8219)	348 (1741)	300 (607)	369 (716)	570 (863)
AUC clumped	0.67 (0.58)	0.61 (0.52)	0.67 (0.60)	0.69 (0.61)	0.71 (0.67)
AUC separated	0.69 (0.59)	0.62 (0.51)	0.68 (0.60)	0.66 (0.61)	0.75 (0.71)

Main values are for analyses of high-confidence GWAS hits. Values in parentheses are for all SNPs in the GWAS Catalogue.

**Figure 4:**
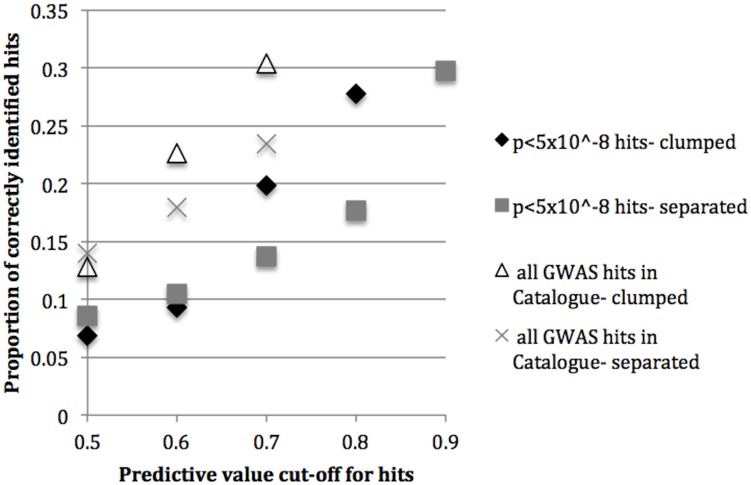
Proportion of correctly identified hits in the test data (positive predictive values). In the non-phenotype specific analyses at various cut-offs for defining hits: SNPs with predictive values of greater than 0.5, 0.6, 0.7, 0.8, or 0.9. Note that results are only plotted for those predictive value thresholds in which there are at least 11 hits correctly identified.

We also investigated positive predictive values (PPVs) and histograms of the probability of causality (prediction score). PPV estimates could not be obtained due to insufficient data (a limited number of true hits correctly identified as hits at a particular prediction value threshold) for the phenotype specific analyses since these analyses contain only a subset of all GWAS hits. As a result, PPVs were only plotted for the non-phenotype specific analyses (**Figure 4**). PPVs appear to be highest for the analysis using all GWAS hits compared to the analysis using the high-confidence hits when defining hits as those variants with a prediction score of greater than 0.5, 0.6, or 0.7. There was insufficient data at the higher thresholds for declaring a positive hit for the analysis based on all GWAS hits. Yet sufficient data was available at the higher prediction value thresholds for the analysis using the subset of high-confidence hits, demonstrating a broader spread in prediction values for that analysis compared to the analysis on all GWAS hits.

**Figure 5:**
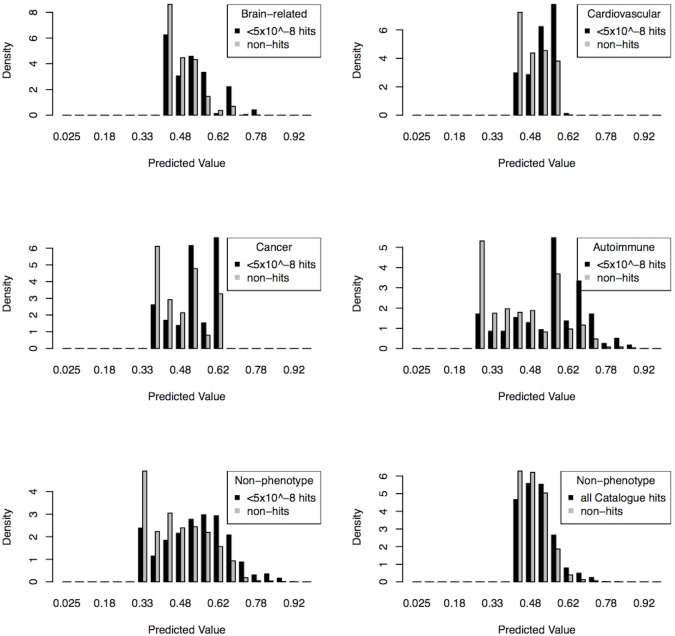
Predicted values for true GWAS hits and non-hits in the test data. Panels show results of clumped-variable analyses on high-confidence GWAS hits for brain-related [**a**], cardiovascular [**b**], cancer [**c**], autoimmune [**d**], and non-phenotype specific hit sets [**e**], and for all hits in the GWAS Catalogue for the non-phenotype specific hit set [**f**].

Histograms of the probability of causality in the test data allowed visualization of the separation (or non-separation) of true hits versus non-hits. We found that for the non-phenotype specific analysis and for the autoimmune analysis, the use of high-confidence GWAS hits in the training data improved the separation of true hits from non-hits in the test data (**Figure 5**).

The results from the histograms of the predicted values showed a broader spread in the non-phenotype specific clumped analysis on high-confidence GWAS hits compared to the analysis using all hits. The former separated true hits from non-hits better than the latter, with the modes of the two distributions distinct. These results suggest that the weighted elastic net procedure was successful in producing models that performed well in identifying true hits as well as in identifying true non-hits. While we could not obtain reliable PPV estimates for the autoimmune analysis due to insufficient data, the separation of non-hits from hits in the histogram was taken as sufficient evidence that the high area under the ROC curve for the autoimmune clumped analysis was also due to positive predictive power.

Results will only be provided for the non-phenotype specific and the autoimmune clumped analyses, the two models that were deemed to be reliable based on the predictive accuracy measures. For the non-phenotype specific clumped analysis, the highest Bayes factor for annotation (11.95) was obtained for rs11177, which is a known GWAS hit associated with osteoarthritis on chromosome 3. It had a predicted value of 0.93. This SNP held all functional characteristics except three low-frequency characteristics: splice sites, miRNA targets, and Gencode transcription start sites. Nine percent of the variants with the top 500 Bayes factors were known GWAS hits. The frequency of hits in the test set data was 4.1%. The mean and median of the predicted values for the true hits in the test set were higher than those for the true non-hits (for hits: mean = 0.54, standard deviation = 0.13 and median = 0.54; for non-hits: mean = 0.46, standard deviation = 0.12 and median = 0.44).

For the autoimmune clumped analysis, the SNP with the highest Bayes factor was the same as for the non-phenotype specific clumped analysis, rs11177.

### Investigation of the Relative Importance of Different Functional Characteristics

**Figure 6:**
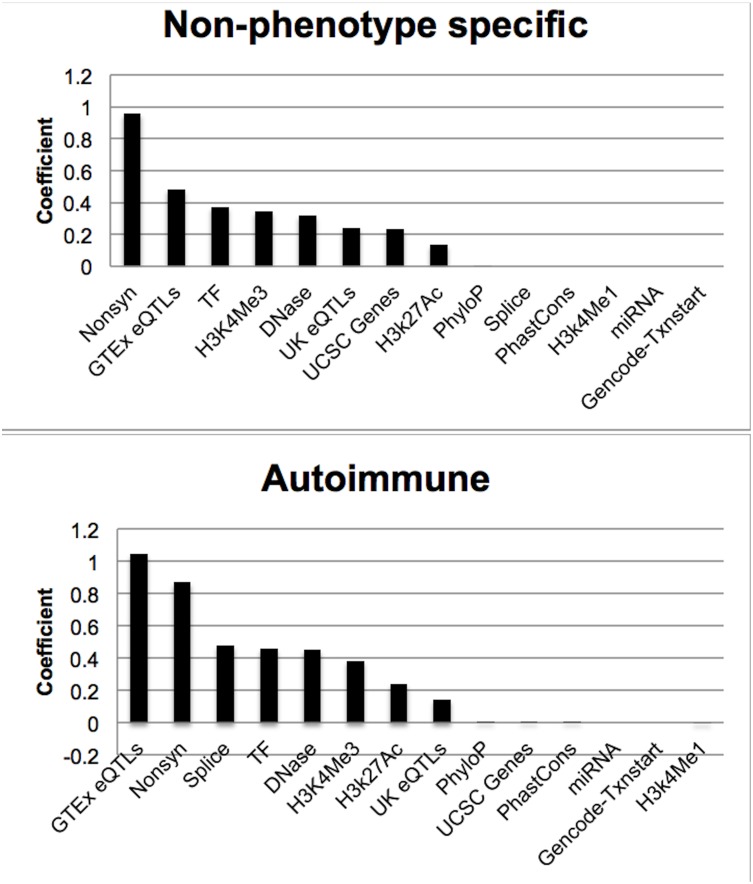
Coefficients of the functional characteristics for the two best analyses. The figure shows the coefficients from the clumped analysis on high-confidence GWAS hits for the non-phenotype specific versus the autoimmune model.

The importance of a particular functional characteristic in predicting whether or not a SNP is more probable to be a GWAS hit is assessed by means of the magnitude of the coefficient assigned to the characteristic. In both the non-phenotype specific and autoimmune analyses we note that the nonsynonymous SNP functional characteristic had one of the highest coefficients (**Figure 6**). (The coefficients for both models are provided in **File S1, Part B**. Confidence intervals cannot be easily calculated for coefficients from elastic net, and so to estimate standard error for the coefficients we performed multivariate logistic regression. Those results are also in **File S1, Part B**.) GTEx eQTLs had the highest coefficient in the autoimmune analysis.

### Investigating Functional Predictions in the Context of known GWAS

We investigated: schizophrenia (SZ) from a meta-analysis GWAS involving the first sample from the Psychiatric Genomics Consortium (PGC1) combined with a Swedish sample [15], systolic blood pressure (SBP) from the International Consortium for Blood Pressure (ICBP) [16], and height from Genetic Investigation of Anthropomorphic Traits (GIANT) Consortium [17]. The studies analyzed over 35,000 cases and 47,000 controls, 200,000 individuals and, and over 180,000 individuals, respectively.

**Figure 7:**
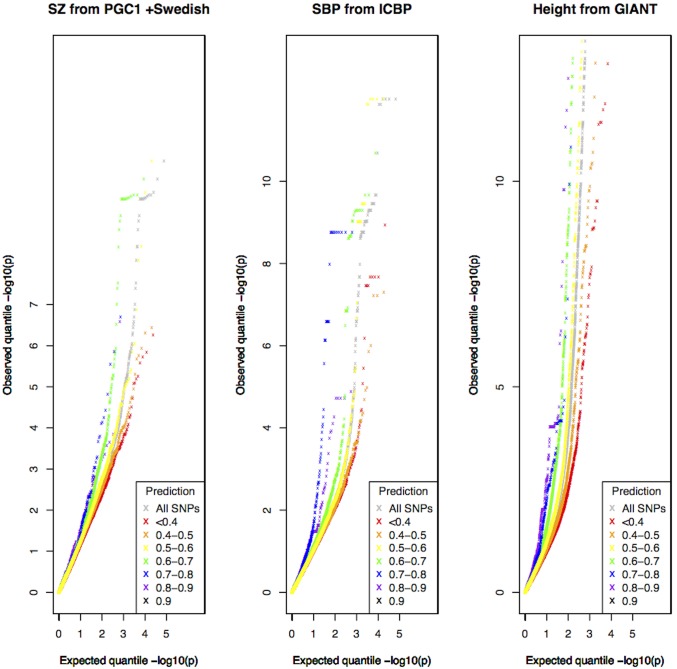
Quantile-quantile plots stratified by predicted values for SNPs in real GWAS. All GWAS SNPs (in grey) for a schizophrenia GWAS from PGC1 with a Swedish sample [**a**], a systolic blood pressure GWAS from ICBP [**b**], and a height GWAS from GIANT [**c**]. The non-grey lines show plots for SNPs binned according to their predicted value from the non-phenotype specific model.

For each study, we stratified the quantile-quantile plots according to predicted value bins (**Figure 7**). We found that SNPs with higher predicted values from the non-phenotype specific clumped analysis tended to deviate more from the line corresponding to the overall GWAS, in favour of more association signals. Similar results were obtained for all three GWAS analyzed: schizophrenia, systolic blood pressure and height. The pattern remained when only the GWAS SNPs present in the test set were plotted, and also when prediction values were obtained from models derived from excluding the genome-wide significant SNPs in the training set for each GWAS respectively. (Results shown in **File S1, Part C**).

We obtained summary data obtained from a psoriasis GWAS study from Strange et al. [18]. We then selected 15 SNPs that were subsequently discovered in a meta-analysis [19]. Using summary association statistics from the Strange et al. study we derived Bayes factors for association (BF_assoc_) and Bayes factors based on association data combined with the annotation of functional characteristics (BF_assoc_*BF_annot_) for each SNP. We ranked the SNPs according to BF_assoc_, and ranked them again according to BF_assoc_*BF_annot_ to determine whether annotating SNPs with their functional characteristics improved their rank (larger Bayes factors were assigned smaller ranks). BF_annot_ values were derived from the non-phenotype specific clumped analysis using high-confidence GWAS hits. As negative controls, we took 12 independent sets of a random 15 SNPs (which were not in high LD with any of the 15 hits and had similar p-values to the hits) and compared the difference in the sum of ranks based on BF_assoc_ versus BF_assoc_*BF_annot_. The procedure was repeated using BF_annot_ derived from the autoimmune clumped analysis.

Of the 15 true psoriasis hit SNPs, 7 had better ranks based on BF_assoc_*BF_annot_ compared to association information on its own (BF_assoc_). The difference of the sum of ranks assigned to the 15 hits was nearly 48,000 based on BF_assoc_*BF_annot_ compared to BF_assoc_, with the former having the lower sum (better ranks). Many of the hit SNPs had very large ranks based merely on the association data (>3000), which was also the case for ranks based on BF_assoc_*BF_annot_, but the trend was in the right direction with better ranks obtained when combing the association information with the annotation of functional characteristics. Of the 12 random sets of 15 independent SNPs, the trend was in the opposite direction for 10 of the sets (with SNPs having better ranks based on BF_assoc_ alone). Of the remaining 2 sets, one of them had the same number of the SNPs with improved ranks based on BF_assoc_*BF_annot_ compared to BF_assoc_ as did the analysis with the actual hits (7 out of 15), and the other random set had 8 SNPs that showed improvement. However, for those random SNP lists the difference in the sum of ranks from BF_assoc_ compared to BF_assoc_*BF_annot_ was less than half of the improvement of ranks seen for the 15 hits. Comparable results were seen when using BF_assoc_ based on the autoimmune clumped analysis. The difference between the sum of the ranks for BF_annot_ compared to BF_assoc_*BF_annot_ was over 49,000, with improved ranks of the hits based on the BF_assoc_*BF_annot_ ranks. Of the random lists the largest difference in the sum of ranks from BF_assoc_ compared to BF_assoc_*BF_annot_ was less than a third of the improvement of ranks seen for the 15 hits.

## Discussion

The release of major genome wide datasets such as ENCODE and NIH Roadmap projects, offers an excellent opportunity to re-assess the existing GWAS corpus and draw conclusions about which functional characteristics in the human genome are most likely to indicate causality in association studies. We previously considered Bayes factors based on a limited set of functional characteristics, considering each functional characteristic separately [3]. Here we have extended our Bayesian framework by developing Bayes factors for multiple functional characteristics, considering all functional characteristics jointly. We used a regularized logistic regression to fit predictive models allowing for large numbers of both qualitative and quantitative functional characteristic data. We performed our analysis under a wide variety of conditions, including phenotype specific analysis for autoimmune, brain-related, cancer, and cardiovascular disorders.

Our results confirm previous findings of differences in functional enrichment in GWAS hits compared to non-hits, which provided a rationale for utilizing functional characteristics as predictors of SNP causality. We found that using high-confidence GWAS hits (p<5×10^−8^) as a classifier resulted in more predictive power. However, if the number of GWAS hits that are available for training are too low, then the predictions become imprecise. This was a reoccurring theme for many of the phenotype specific analyses. The separation between true GWAS hits and non-hits in the test set, in addition to the AUC, should be used to assess the predictive power of a model. Using those methods we found that the non-phenotype specific and the autoimmune analyses on clumped variables using high-confidence GWAS hits were most reliable. For instance, although the AUCs were slightly higher for the separated analyses, the classification of true GWAS hits and non-hits was better in the clumped analysis, suggesting that the clumped analysis may provide more accurate predictions. The benefit of the separated analysis is that it allows researchers to identify characteristics specific to certain conditions, for example specific cell types, which can be useful for planning further investigations, but the increased number of variables and sparsity of the data reduces the power of this type of analysis.

While our study has demonstrated that relevant functional information is indeed predictive for identifying GWAS hits, and that Bayes factors incorporating this functional information rank known GWAS hits better than Bayes factors based on association information alone, the improvements based on current information (for example, in the psoriasis GWAS we analyse) are marginal. However, we outline reasons below to argue that the benefit of adding functional information to analyses of causal variant discovery will increase in the future.

A limitation to the study is the restricted amount of tissue- or cell-specific data, especially in light of the findings that enrichment of disease-specific GWAS hits can differ in certain cell types, for example for DNase I hypersensitive sites [8]. Incorporating additional functional characteristics, for example those from relevant tissue types, will likely improve the understanding of which characteristics are associated with GWAS hit SNPs, especially for the phenotype specific analyses. Furthermore, other functional characteristics, such as further histone marks and other epigenetic modifications, could be incorporated to improve the models.

The current number of GWAS hits in the GWAS Catalogue makes it challenging to sub-divide hits into phenotype specific traits. However, preliminary results showing differences in the coefficients for the functional characteristics suggest that as the number of GWAS hits grows, a phenotype specific approach from which to derive Bayes factors for prioritization could be more biologically relevant than simply an approach that combines all GWAS hits together. Interestingly, although it was one of the largest lists, the brain-related list did not have a greater predictive power than expected by chance. This finding only serves to reinforce the widely appreciated complexity of brain-related disorders. Nevertheless, schizophrenia GWAS significant SNPs showed enrichment of SNPs with high predicted values from the model, as did SNPs associated with systolic blood pressure or height.

Using manually curated phenotype lists as done here may not be the best option. Using lists that are more reproducible, such as those based on the Experimental Factor Ontology (EFO) definitions, may be more appealing. However, most of the lists created using the EFO definitions were relatively small, covering less than 10% of the total GWAS hits on the common genotyping arrays, and thus this method of classifying GWAS hits was deemed to be not feasible, but may be possible in the future as the size of GWAS Catalogue grows still larger.

The coefficient for non-synonymous SNPs was the highest in the non-phenotype specific analysis and a close second in the autoimmune analysis. This result suggests that being a variant in a gene that causes a protein alteration is an important indicator of whether or not a genetic variant will be truly associated with a phenotype. The result agrees with the findings that the top associated SNPs and also those that are nominally associated with a phenotype are more likely to overlap genes than non-GWAS SNPs [20]. Our analysis appears to underscore the primacy of non-synonymous variation as a leading mediator of functional variation in the human genome. Although this result is perhaps unsurprising, it lends support to many of the gene-focused, rare-variant strategies that have been recently employed (for example: [21], [22], [23]. However, depending on the inclusiveness of promoter regions in chip design, these strategies may or may not capture other high scoring variant types, such as eQTLs and histone marks, which collectively account for more GWAS hits than non-synonymous variants alone. These patterns highlight a possible need for follow-up on non-coding variation chips. GTEx eQTLs came up as the most important factor in the autoimmune analysis. Two of the experiments analyzed eQTLs from lymphoblastoid cells, which may explain the importance of this functional characteristic in the autoimmune traits.

We have shown that our method can be used to calculate Bayes factors for annotation (BF_annot_). These can be applied to GWAS data to prioritise near-significant variants for follow-up based on the likelihood of being causal in light of their functional characteristics. The method takes LD into account, and uses information from the March 2012 release of the 1000 Genomes Project to map relevant annotation information from all variants in high LD, including both SNPs and indels. In addition to being used for variant prioritization of GWAS data, the methodology could be applied in the future to the prioritization of variants from fine mapping and sequencing studies. Here, the question arises as to whether the models described here, which were created based on common variation, could be applied to rare variation. In time, larger databases of true causal variation, including rare variation, will allow our method to be applied with increasing accuracy.

## Methods

### Representative GWAS SNPs

To represent the characteristics of a typical GWAS panel, markers from the Affymetrix Genome-Wide Human SNP Array 6.0, the Illumina Human1M–Duo Genotyping BeadChip, and the Illumina HumanOmni1-Quad BeadChip were downloaded from the UCSC genome browser, using the table browser tool [24]. The union of these three arrays consisted of 1,936,864 unique SNPs from the 22 autosomes. Because of its unique LD and genic properties, the MHC region (chr6: 29624809–33160245 on build 37) was excluded from downstream analyses.

LD proxies or “tagging” SNPs (r^2^> = 0.8) for the GWAS panel SNPs were identified using VCFtools [25] based on data from the (N = 379) Europeans (Phase I, version 3, March 14, 2012) in the 1000 Genomes Project [26].

GWAS “non-hits” were defined as all those SNPs in our union GWAS set which were neither a GWAS “hit” (see below), nor in high LD (r^2^> = 0.8) with a GWAS hit.

### GWAS Hits

To obtain a set of SNPs (and their LD proxies) with good prior evidence of causality, we downloaded the Catalogue of Published Genome-wide Association Studies from the National Human Genome Research Institute (NHGRI) (http://www.genome.gov/gwastudies) [1] on August 6, 2013. This catalogue contains a list of SNPs that have been shown to be associated with a particular trait in a GWAS at a suggestive p-value<10^−5^. We removed SNPs in the Catalogue that were not present in the representative GWAS set defined above, and similarly removed SNPs on the sex chromosomes or in the MHC region.

All SNPs in our GWAS hit and GWAS non-hit sets, along with all their LD proxies, were annotated with all the functional characteristics defined below. Each GWAS hit and non-hit SNP was then given the maximum value for each functional characteristic found across all its LD proxies.

### Functional Characteristics

We acquired functional data from a variety of sources (**Table 4**). A full list is provided in **Table S1**. Much of the data was downloaded from the UCSC genome browser using the table browser tool [24]. Additionally, a substantial proportion of the data was derived from the ENcyclopedia of DNA Elements (ENCODE) Project Consortium, which developed and implemented a range of experimental techniques with the aim of identifying the functional regions of the human genome, particularly including non-coding regions [27]. Data from this project that were used included transcription factor binding sites (TFBSs), three histone modifications (H3K4Me1, H3K4Me3, H3K27Ac), and DNase I hypersensitive sites. H3K4Me1 is associated with enhancers and DNA regions downstream of transcription starts, and often found near regulatory elements; H3K4Me3 is associated with promoters active or poised to be active, and often found near promoters; H3K27Ac thought to enhance transcription possibly by blocking repressive histone mark H3K27Me3, and often found near active regulatory elements. The technologies for identifying the functional characteristics mentioned above were chromatin immunoprecipitation followed by sequencing (ChIP-seq).

**Table 4:** Summary of functional characteristics.

Functional characteristic analysed	Description	Number and detail of measures used in the analysis*	
		Clumped	Separated
**ENCODE data**			
UW DNase I hypersensitive sites	Data from digitalDNaseI methodology,Replication 1 samples; (“peaks”)	N/A	122
Duke DNase I hypersensitive sites	Positions of open chromatinby FAIRE and ChIP-seqexperiments; (“peaks”)	N/A	100
DNase Clusters (v2)**	Stringent (FDR 1% threshold)for “peaks” of DNaseI hypersensitivity fromuniform processing by theENCODE Analysis WorkingGroup of data from UW and Duke	1	N/A
Txn Factor ChIP	Transcription factorbinding sites (TFBS)from ChIP Seqexperiments; (“peaks”)	1 (presence or absencein any TFBS)	148 (separated by TF, but not by celltype due tosparse data)
Broad Histone – H3K4Me1, H3K4Me3, H3K27Ac	All are assayed using ChIP-Seq; (“peaks”)	3 (each histone markgrouped by the 18cell typesand/or conditions)	54 (each histone mark separatedby cell typeand/or conditions)
**Conservation**			
PhyloP	Average scores can be calculatedas the sum of scores dividedby the number of valid datavalues in the block(scores range from 0.1 to 2.2910)	1	1
PhastCons	Average scores can becalculated as for PhyloP(scores range from 0.1 to 1.0 in this dataset)	1	1
**Expression quantitative trait loci**			
eQTL- GTEx	cis-eQTLs, p<1×10^−5^ cut-off for variantswithin 2 Mb of theexpressed gene.	1 (any eQTL)	7 (separated by dataset)
eQTLs - UK Brain	cis-eQTLs, FDR<1% cut-off for variantswithin 2 Mb of theexpressed gene.	1	1
**Other characteristics**			
UCSC Genes	UCSC known Gene	1	1
Splice sites	Splice site region definedas −5 to +5 range aroundexon starts & exon endsof UCSC Genes	1	1
Nonsynonymous SNPs	Coding Nonsynonymous SNPs definedas stop-gain(nonsense), missense,stop-lost, frameshift orinframe indel	1	1
TS miRNA sites	Conserved mammalian microRNA regulatorytarget sites forconserved microRNAfamilies	1	1
GencodeTranscriptionstart sites	Based on theGENCODEGenes variable (version 17, June 2013)	1	1

*All SNPs are annotated in a binary fashion indicating the presence or absence of a functional characteristic, except for the conservation scores, for which the SNPs are assigned a quantitative score.

**The DNase Clusters v2 file was created by combining the UW and Duke DNase I data that have been uniformly processed and replicates merged. Stringent (FDR 1% thresholded) peaks of DNase I hypersensitivity from uniform processing by the ENCODE Analysis Working Group were applied. Grouping the UW and the Duke DNase I hypersensitive variables are not equivalent to the DNase Clusters v2 file, and thus we used the latter to represent DNase I hypersensitive sites in the clumped analysis due to the substantial efforts made to combine the data meaningfully.

DNase I hypersensitive sites are regions in the genome with high affinity of being cleaved by the DNase I enzyme. The University of Washington (UW) group identified DNase I hypersensitive sites using Digital DNase I. This method involves DNase I digestion of intact nuclei, isolation of DNaseI “double-hit” fragments, and direct sequencing of fragment ends. Peaks are regions that are enriched in the captured fraction of the DNA suggesting they are occupied by the protein of interest (any score >0). The DNase I hypersentitive sites from the Duke University group were identified using a synthesis of Formaldehyde-Assisted Isolation of Regulatory Elements (FAIRE) and ChIP-seq experiments. We used a binary variable to indicate whether a SNP was within a peak.

Two types of conservation scores from 46 placental mammals (PhyloP and PhastCons) were incorporated. Both PhyloP and PhastCons scores are derived using phylogenetic hidden Markov models. These two measures have their own advantages. PhyloP scores do not take into account conservation at neighbouring sites, whereas PhastCons estimates the probability that each nucleotide belongs to a conserved element.

Expression quantitative trait loci (eQTLs), which are variants that are correlated with gene expression, were included. In particular those that fall within 2 Mb (+/−1 Mb upstream and downstream) (cis-eQTLs) of the gene of interest were used. These data were derived from the NCBI-hosted GTEx Browser (http://www.ncbi.nlm.nih.gov/gtex/GTEX2/gtex.cgi) [28], [29], [30], [31] and the UK Brain Expression Consortium (www.braineac.org) [32].

Summary information concerning the location or function within a gene (coding-non-synonymous, coding-synonymous, splice site, untranslated regions, etc) was derived from dbSNP. Non-synonymous SNPs, were classified as those SNPs with one of the following characteristics: stop-gain (nonsense), missense, stop-lost, frameshift or inframe indel. Splice site regions were defined as being within five base pairs upstream and five base pairs downstream of the exon start site or the exon end site. The UCSC gene table was used to determine the exon start and end sites. The UCSC gene table is comprised of a set of gene predictions based on data from RefSeq, GenBank, the Consensus Coding Sequence (CCDS) variable, Rfam, and the Transfer RNA Genes variable. Additional characteristics used were 3′ targets for microRNA (miRNA), and also transcription start sites as described by Gencode [33]. As miRNA targets are known to be substantially over-predicted, we used a conservative miRNA target dataset based on conserved mammalian microRNA regulatory target sites in the 3′ UTR regions of Refseq Genes, as predicted by the TargetScan algorithm (Human 5.1) [34].

All SNPs in our GWAS hit and GWAS non-hit sets, along with all their LD proxies, were annotated with all the functional characteristics defined above. Each GWAS hit and non-hit SNP was then given the maximum value for each functional characteristic found across all its LD proxies.

### Tests for Functional Enrichment

Counts of GWAS hits and non-hits were categorized by annotation value and compared using Fisher’s exact test. To verify that results were not unduly influenced by correlations (LD) among observations, we also conducted analyses in which genetic variants were “pruned” so that all SNPs have r^2^<0.8 with all other SNPs. The results of these analyses were very similar (data not shown).

Heat maps were constructed using R [35] to compare correlations among the various functional characteristics.

### Regularized Logistic Regression via Elastic Net

We used a regularized form of logistic regression known as elastic net to predict GWAS hit versus non-hit status on the basis of the functional characteristics we had collected. We first employed this method for a symposium on “Functional annotation of GWAS hits” that we organized for the American Society of Human Genetics in 2010. Elastic net is a form of machine learning first described by Zou and Hastie [36], and is implemented in the glmnet package [37] in R [35]. Briefly, regularization is achieved via the subtraction of a penalty term from the log-likelihood prior to maximization. The penalty term includes both a “lasso-like” L1 component (the sum of the absolute values of all fitted coefficients) and a “ridge-like” L2 component (the sum of squares of all fitted coefficients). Two parameters, alpha and lambda, determine the relative importance of the L1 versus the L2 term (alpha), and the overall importance of the penalty term in the maximization (lambda). Appropriate values for these parameters were found by 10-fold cross-validation of the training set (see below).

Due to the unbalanced nature of the data (many more GWAS non-hits than hits) we employed a weighting procedure in the logistic regression to balance the accuracy of prediction in both types of markers. We weighted all hits by (N_hits_+N_non-hits_)/2N_hits_ and all non-hits by (N_hits_+N_non-hits_)/2N_non-hits_, where N_hits_ and N_non-hits_ denote the number of hits and non-hits, respectively, in the training set. This procedure has the effect of equalizing the importance of hits and non-hits in the logistic regression.

We randomly selected 60% of our GWAS hits and non-hits to form our training set. The remaining 40% of the data (the test set) was used to assess the performance of the model using ROC curves and other measures. We repeated the machine learning modifying the percentage of the data used in the training and test sets, and all splits produced similar results (**File S1, Part D**). To diminish the possibility that the models are over-fit since the training of the data and tuning of the parameters were conducted on the same set, we created a 70%/30%, split where the 70% was further split into 60% and 40% for training the coefficients and tuning the parameters, respectively. The remaining 30% was used to test the model. Similar results were produced when the training and tuning were conducted in independent subsets. (**File S1, Part D**), and so the 60%/40% training/test set split was pursued for the remaining analyses.

The data was split into the training and test sets ten times using a random number generator. We found that the beta coefficients were consistent for all of the functional characteristics with the exception of those with the lowest frequencies (**File S1, Part E**).

For the calculation of Bayes factors, we performed elastic net, using the same determined values of alpha and lambda, on the full GWAS hit and non-hit datasets.

### Predictive Accuracy

We employed three methods to determine which models had the best predictive accuracy: ROC curves, positive predictive values, and histograms of the predicted values from the models.

ROC curves show the sensitivity and specificity of a fitted model. Sensitivity is the probability of the model providing a true positive result (identifying a true GWAS hit in the test set). Specificity is the probability of the model providing a true negative result (identifying a true GWAS non-hit in the test set). An AUC of 0.5 indicates a model of no predictive value, while an AUC of 1 indicates perfect predictive power. The ROC curves were created using the ROCR package [38] in R.

ROC curves do not reflect how well a model performs within each class given unbalanced data (a very large number of non-hit SNPs compared to hits). To capture this aspect we also investigated positive predictive values (PPVs), the proportion of SNPs with predicted probabilities of causality above a certain threshold (we investigated thresholds of 0.5, 0.6, 0.7, 0.8 or 0.9) that are true GWAS hits in the test set. Finally, we visualized class separation with histograms of the predicted probabilities of causality by class.

### Definition of Functional Variables and GWAS Hits

A variety of functional characteristics were investigated as input variables. One, defined as the “clum ped” analysis, featured groups of functional characteristics, which were collapsed into a single summary variable. The “separated” analysis worked on all functional characteristics individually.

We performed phenotype specific analyses in which the analyses outlined above were carried out using phenotype specific GWAS hits as classifiers. An autoimmune list, a brain-related list and a cardiovascular list were created using the GWAS Catalogue searching for terms relating to those phenotypes. Each list was then verified by an expert in the field.

Additionally, the GWAS Catalogue was divided up into categories specified by the Experimental Factor Ontology (EFO) definitions; however, due to small numbers of SNPs in each category this mode of classification is not currently feasible for most of the subsets (**File S1, Part F**). Only the cancer list, which was the largest disease-relevant list, was used.

We defined two sets of GWAS hits for downstream analysis, one based on a weak significance threshold of p<10^−5^ and one based on a strong significance threshold of p<5×10^−8^, as reported in the NHGRI GWAS Catalogue.

### Derivation of Bayes Factors

Bayesian analysis provides the most suitable framework for combining functional characteristics (here referred to as “annotation data”), with evidence from an association study (“association data”) [39]. We expand on our previous empirically-based approach to the calculation of Bayes factors for annotation [3] to allow multiple functional characteristics to be considered simultaneously. The posterior odds (O_post_) of causality for a trait of interest at a given SNP are given by the ratio of the conditional probability of causality, given the annotation and the association data, to the conditional probability of non-causality:
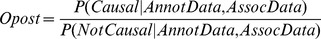



If we assume the annotation data and association data are independent once conditioned on causality, then the posterior odds become:
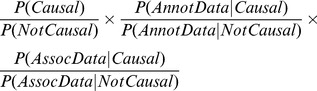



These three products are, respectively, the prior odds before seeing any association and annotation data (O_prior_), the Bayes factor for annotation data (BF_annot_) and the Bayes factor for association data (BF_assoc_). We note that this factorization implies that, while functional annotations are allowed to be enriched (or impoverished) for causal SNPs relative to non-causal SNPs, the enrichment pattern is assumed to be the same for rare versus common causal SNPs, and for low-effect size versus high effect size causal SNPs. We accept that this is an imperfect approximation, and it assumes among other things that SNPs are either causal or non-causal when in reality their effect size can be arbitrarily close to zero, but we note that the main limitation of our approach lies with the small number of GWAS hits available to us, and subdividing these still further according to allele frequency and effect size would be problematic. We also note that by “causal” what we actually mean is “causal or in high LD with a causal variant”, as both the association data and the annotation data (as defined in our study) are affected by LD proxies.

In our previous study [3], we noted that if one assumed that (1) all hits in the NHGRI GWAS Catalogue were truly causal; and (2) functional annotation enrichment patterns were the same for these known hits as for future undiscovered truly causal SNPs; then an empirically based estimate for BF_annot_ for a single binary functional characteristic would simply be the ratio of its frequency in GWAS hit versus non-hit data. Here we note that if we start with the same two assumptions, and further assume that a true (but unknown) logistic model exists that relates a set of functional characteristics (which can be either binary or quantitative) to the probability that a SNP is truly causal, then one reasonable approach to estimating that logistic model would be via regularized logistic regression as described above. Once fitted, the estimated odds of causality to non-causality, obtained from the GWAS hit and non-hit datasets, need only be multiplied by the prior odds of non-causality in these dataset (i.e. the ratio of the weighted sample sizes of GWAS non-hits to GWAS hits in these data) in order to obtain the Bayes factor for annotation. Here, we chose to weight hits and non-hits to appear of equal size, and thus our estimate for BF_annot_ is obtained directly as the estimated odds of causality to non-causality from the regularized logistic regression.

Methods for estimating BF_assoc_ from association data are reviewed by Stephens & Balding [39]. Here, we use the convenient approximation described by Wakefield [40].

### Investigating the Model in the Context of known GWAS

To investigate the relevance of the predictions in a variety of disorders we looked at the p-value distribution of SNPs according to their functional class in large GWAS datasets with a substantial fraction of GWAS significant findings. Quantile-quantile plots were constructed for each study with multiple lines corresponding to SNPs binned according to their predicted value. Predicted values were those derived from the non-phenotype specific clumped model in which GWAS hits were defined as those SNPs in the GWAS Catalogue with p-values of less than 5×10^−8^. We expected those SNPs with higher predicted values to be enriched with GWAS SNPs with more significant p-values, whereas those SNPs with lower predicted values would be enriched with less significant p-values compared to all SNPs in the GWAS.

We also selected some SNPs shown to be associated in a large psoriasis meta-analysis which had not been identified in a previous GWAS study [18], [19]. We then determined the effect on the rank of their Bayes Factors in the previous study derived either using association data or both association data and functional characteristics.

## Supporting Information

Table S1 Full list of functional characteristics utilized in the separated analyses.
(XLSX)

File S1 **Supporting Information- Parts A–F.**
(PDF)

## References

[1] Hindorff LA, Junkins HA, Hall PM, Mehta JP, Manolio TA (2010). A catalog of published genome-wide association studies. Available at: www.genome.gov/gwastudies. Accessed 2013 August 6.

[2] Purcell SM, Wray NR, Stone JL, Visscher PM, O’Donovan MC, et al (2009) Common polygenic variation contributes to risk of schizophrenia and bipolar disorder. Nature 460: 748–752. 19571811 10.1038/nature08185 PMC3912837

[3] Knight J, Barnes MR, Breen G, Weale ME (2011) Using functional annotation for the empirical determination of Bayes Factors for genome-wide association study analysis. PLoS One 6: e14808. 21556132 10.1371/journal.pone.0014808 PMC3083387

[4] Hindorff LA, Sethupathy P, Junkins HA, Ramos EM, Mehta JP, et al (2009) Potential etiologic and functional implications of genome-wide association loci for human diseases and traits. Proc Natl Acad Sci U S A 106: 9362–9367. 19474294 10.1073/pnas.0903103106 PMC2687147

[5] Nicolae DL, Gamazon E, Zhang W, Duan S, Dolan ME, et al (2010) Trait-associated SNPs are more likely to be eQTLs: annotation to enhance discovery from GWAS. PLoS Genet 6: e1000888. 20369019 10.1371/journal.pgen.1000888 PMC2848547

[6] Bernstein BE, Birney E, Dunham I, Green ED, Gunter C, et al (2012) An integrated encyclopedia of DNA elements in the human genome. Nature 489: 57–74. 22955616 10.1038/nature11247 PMC3439153

[7] Schaub MA, Boyle AP, Kundaje A, Batzoglou S, Snyder M (2012) Linking disease associations with regulatory information in the human genome. Genome Res 22: 1748–1759. 22955986 10.1101/gr.136127.111 PMC3431491

[8] Maurano MT, Humbert R, Rynes E, Thurman RE, Haugen E, et al (2012) Systematic localization of common disease-associated variation in regulatory DNA. Science 337: 1190–1195. 22955828 10.1126/science.1222794 PMC3771521

[9] Boyle AP, Hong EL, Hariharan M, Cheng Y, Schaub MA, et al (2012) Annotation of functional variation in personal genomes using RegulomeDB. Genome Res 22: 1790–1797. 22955989 10.1101/gr.137323.112 PMC3431494

[10] Ward LD, Kellis M (2012) HaploReg: a resource for exploring chromatin states, conservation, and regulatory motif alterations within sets of genetically linked variants. Nucleic Acids Res 40: D930–934. 22064851 10.1093/nar/gkr917 PMC3245002

[11] Dayem Ullah AZ, Lemoine NR, Chelala C (2012) SNPnexus: a web server for functional annotation of novel and publicly known genetic variants (2012 update). Nucleic Acids Res 40: W65–70. 22544707 10.1093/nar/gks364 PMC3394262

[12] Schork AJ, Thompson WK, Pham P, Torkamani A, Roddey JC, et al (2013) All SNPs are not created equal: genome-wide association studies reveal a consistent pattern of enrichment among functionally annotated SNPs. PLoS Genet 9: e1003449. 23637621 10.1371/journal.pgen.1003449 PMC3636284

[13] Darnell G, Duong D, Han B, Eskin E (2012) Incorporating prior information into association studies. Bioinformatics 28: i147–153. 22689754 10.1093/bioinformatics/bts235 PMC3371867

[14] Kindt AS, Navarro P, Semple CA, Haley CS (2013) The genomic signature of trait-associated variants. BMC Genomics 14: 108. 23418889 10.1186/1471-2164-14-108 PMC3600003

[15] Ripke S, O’Dushlaine C, Chambert K, Moran JL, Kahler AK, et al (2013) Genome-wide association analysis identifies 13 new risk loci for schizophrenia. Nat Genet 45: 1150–1159. 23974872 10.1038/ng.2742 PMC3827979

[16] Ehret GB, Munroe PB, Rice KM, Bochud M, Johnson AD, et al (2011) Genetic variants in novel pathways influence blood pressure and cardiovascular disease risk. Nature 478: 103–109. 21909115 10.1038/nature10405 PMC3340926

[17] Lango Allen H, Estrada K, Lettre G, Berndt SI, Weedon MN, et al (2010) Hundreds of variants clustered in genomic loci and biological pathways affect human height. Nature 467: 832–838. 20881960 10.1038/nature09410 PMC2955183

[18] Strange A, Capon F, Spencer CC, Knight J, Weale ME, et al (2010) A genome-wide association study identifies new psoriasis susceptibility loci and an interaction between HLA-C and ERAP1. Nat Genet 42: 985–990. 20953190 10.1038/ng.694 PMC3749730

[19] Tsoi LC, Spain SL, Knight J, Ellinghaus E, Stuart PE, et al (2012) Identification of 15 new psoriasis susceptibility loci highlights the role of innate immunity. Nat Genet 44: 1341–1348. 23143594 10.1038/ng.2467 PMC3510312

[20] Tang CS, Ferreira MA (2012) GENOVA: gene overlap analysis of GWAS results. Stat Appl Genet Mol Biol 11: Article. 10.1515/1544-6115.1784 22499700

[21] Barrans JD, Liew CC (2006) “Chip”ping away at heart failure. Methods Mol Med 126: 157–169. 16930011 10.1385/1-59745-088-X:157

[22] Cortes A, Brown MA (2011) Promise and pitfalls of the Immunochip. Arthritis Res Ther 13: 101. 21345260 10.1186/ar3204 PMC3157635

[23] Center for Statistical Genetics (2012) MetaboChip SNP details. University of Michigan.

[24] Karolchik D, Hinrichs AS, Furey TS, Roskin KM, Sugnet CW, et al. (2004) The UCSC Table Browser data retrieval tool. Nucleic Acids Res. 32(Database issue): D493–6. 10.1093/nar/gkh103 PMC308837 14681465

[25] Danecek P, Auton A, Abecasis G, Albers CA, Banks E, et al (2011) The variant call format and VCFtools. Bioinformatics 27: 2156–2158. 21653522 10.1093/bioinformatics/btr330 PMC3137218

[26] Abecasis GR, Altshuler D, Auton A, Brooks LD, Durbin RM, et al (2010) A map of human genome variation from population-scale sequencing. Nature 467: 1061–1073. 20981092 10.1038/nature09534 PMC3042601

[27] The ENCODE Project Consortium (2011) A user’s guide to the encyclopedia of DNA elements (ENCODE). PLoS Biol 9: e1001046. 21526222 10.1371/journal.pbio.1001046 PMC3079585

[28] Montgomery SB (2010) Transcriptome genetics using second generation sequencing in a Caucasian population. Nature 464(7289): 773–777. 20220756 10.1038/nature08903 PMC3836232

[29] Schadt EE (2008) Mapping the genetic architecture of gene expression in human liver. PLoS Biol 6(5): e107. 18462017 10.1371/journal.pbio.0060107 PMC2365981

[30] Gibbs JR (2010) Abundant Quantitative Trait Loci Exist for DNA Methylation and Gene Expression in Human Brain. PLoS Genetics 6(5): e1400952. 10.1371/journal.pgen.1000952 PMC2869317 20485568

[31] Stranger B, Forrest M, Dunning M, Ingle C, Beazley C, et al (2007) Relative impact of nucleotide and copy number variation on gene expression phenotypes. Science 315: 848–853. 17289997 10.1126/science.1136678 PMC2665772

[32] Trabzuni D, Ryten M, Walker R, Smith C, Imran S, et al (2011) Quality control parameters on a large dataset of regionally dissected human control brains for whole genome expression studies. J Neurochem 119: 275–282. 21848658 10.1111/j.1471-4159.2011.07432.x PMC3664422

[33] Harrow J, Frankish A, Gonzalez JM, Tapanari E, Diekhans M, et al (2012) GENCODE: the reference human genome annotation for The ENCODE Project. Genome Res 22: 1760–1774. 22955987 10.1101/gr.135350.111 PMC3431492

[34] Lewis BP, Burge CB, Bartel DP (2005) Conserved seed pairing, often flanked by adenosines, indicates that thousands of human genes are microRNA targets. Cell 120: 15–20. 15652477 10.1016/j.cell.2004.12.035

[35] R Development Core Team (2008) R: A language and environment for statistical computing. R Foundation for Statistical Computing, Vienna, Austria. ISBN 3-900051-07-0, URL http://www.R-project.org/.

[36] Zou H, Hastie T (2005) Regularization and variable selection via the elastic net. Journal Of The Royal Statistical Society Series B-Statistical Methodology 67: 301–320.

[37] Friedman J, Hastie T, Tibshirani R (2010) Regularization Paths for Generalized Linear Models via Coordinate Descent. J Stat Softw 33: 1–22. 20808728 PMC2929880

[38] Sing T, Sander O, Beerenwinkel N, Lengauer T (2005) ROCR: visualizing classifier performance in R. Bioinformatics. 21: 3940–3941. 10.1093/bioinformatics/bti623 16096348

[39] Stephens M, Balding DJ (2009) Bayesian statistical methods for genetic association studies. Nat Rev Genet 10: 681–690. 19763151 10.1038/nrg2615

[40] Wakefield J (2007) A Bayesian measure of the probability of false discovery in genetic epidemiology studies. Am J Hum Genet 81: 208–227. 17668372 10.1086/519024 PMC1950810

