# Supplementary material for: A Bayesian Method to Incorporate Hundreds of Functional Characteristics with Association Evidence to Improve Variant Prioritization
Source: PLoS One. 2014 May 20;9(5):e98122. doi: 10.1371/journal.pone.0098122 (PMC4028284; doi:10.1371/journal.pone.0098122)
Supplement: File S1 — Supporting Information- Parts A–F. (PDF) [file pone.0098122.s002.pdf]

Supplementary Information

Part A- Models not over fit as demonstrated by similar ROC curves in training and test sets

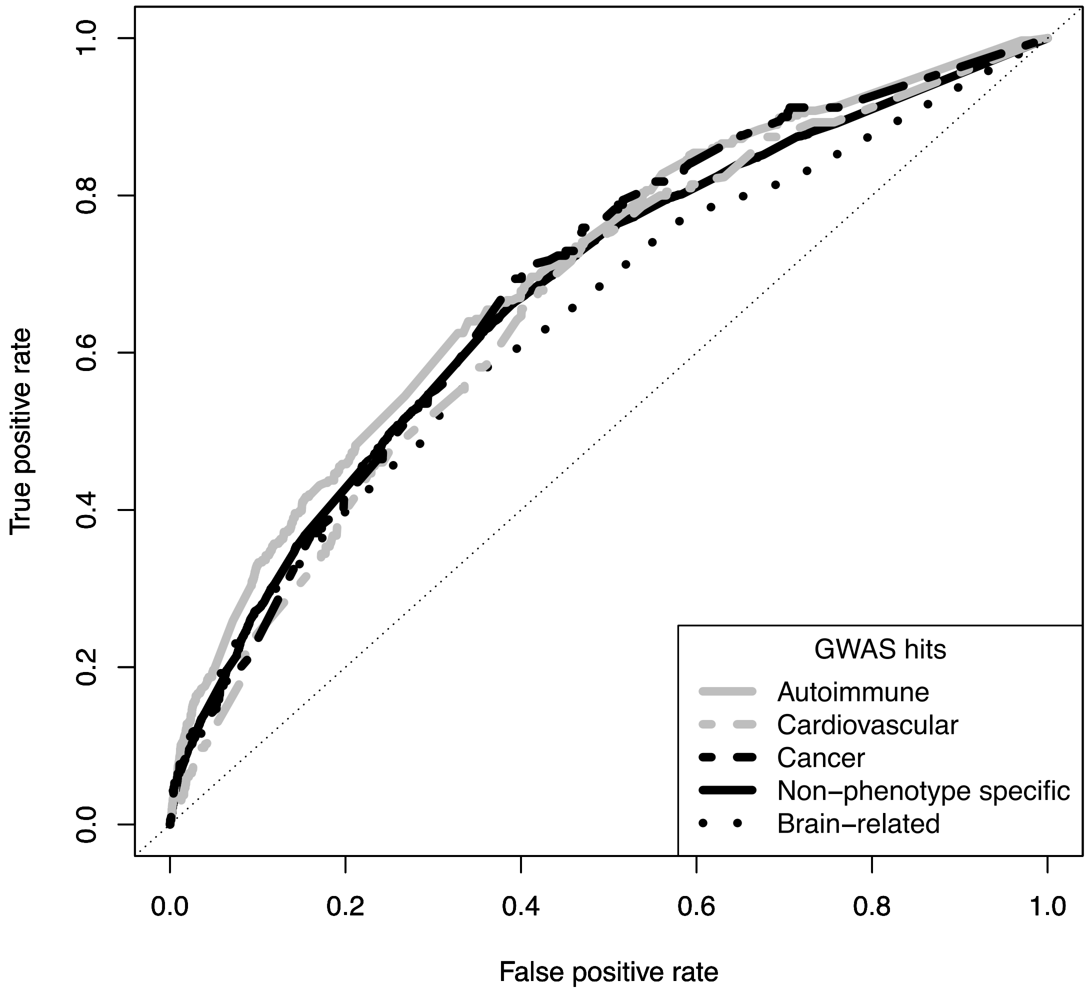

Figure S1. Receiver-operating characteristic (ROC) curves for analyses of clumped functional variables and high-confidence GWAS hits using the training set. This plot is similar to the plot obtained from the separate test set, **Figure 3**.

Part B- Coefficients assigned to the functional characteristics

Table A. **Coefficients for the best two models.** Coefficients for the non-phenotype specific and autoimmune-specific analyses defining hit SNPs as those SNPs in the GWAS Catalogue with a p-value of less than $5x10^{-8}$

	Non-phenotype specific	Autoimmune
Splice	0	0.4787
Nonsynonymous	0.9587	0.8692
DNase I	0.3196	0.4513
GTEx eQTLs	0.4848	1.0452
UK Brain eQTLs	0.2409	0.1420
UCSC Genes	0.2318	0.0012
PhyloP	0.0015	0.0020
PhastCons	0	0.0002
H3k4Me1	0	-0.0062
H3K4Me3	0.3441	0.3832
H3k27Ac	0.1337	0.2382
TFBS	0.3731	0.4590
miRNA	0	0
Gencode-Txnstart	0	0

Table B. **Coefficients and standard error for two models from multivariate logistic regression.** Coefficients and their respective standard errors for the non-phenotype specific and autoimmune-specific analyses defining hit SNPs as those SNPs in the GWAS Catalogue with a p-value of less than $5x10^{-8}$ using multivariate logistic regression

	Non-phenotype specific		Autoimmune	
	Coefficient	Standard error	Coefficient	Standard error
Splice	-1.914	-3.45E-02	1.261	8.23E-03
Nonsynonymous	32.708	2.62E-01	8.857	2.71E-02
DNase I	13.702	3.10E-02	7.281	5.89E-03
GTEx eQTLs	14.024	1.34E-01	7.848	2.70E-02
UK Brain eQTLs	9.602	2.84E-02	3.293	3.50E-03
UCSC Genes	5.39	9.48E-03	-0.421	-2.64E-04
PhyloP	7.502	1.98E-04	0.204	1.92E-06
PhastCons	0.075	1.86E-06	1.413	1.25E-05
H3k4Me1	-3.59	-8.87E-03	-2.553	-2.25E-03
H3K4Me3	8.878	2.06E-02	4.797	3.95E-03
H3k27Ac	3.267	8.16E-03	2.257	2.01E-03
TFBS	10.22	1.88E-02	5.14	3.36E-03
miRNA	0.103	7.77E-03	-0.508	-1.39E-02
Gencode-Txnstart	-0.495	-4.23E-02	-0.816	-2.54E-02

Part C- Application to real GWAS: similar results when (1) only GWAS SNPs present in the test set were plotted and (2) when using prediction values derived from models that were re-run for each GWAS with the removal of the significant SNPs found in that particular GWAS from the training set

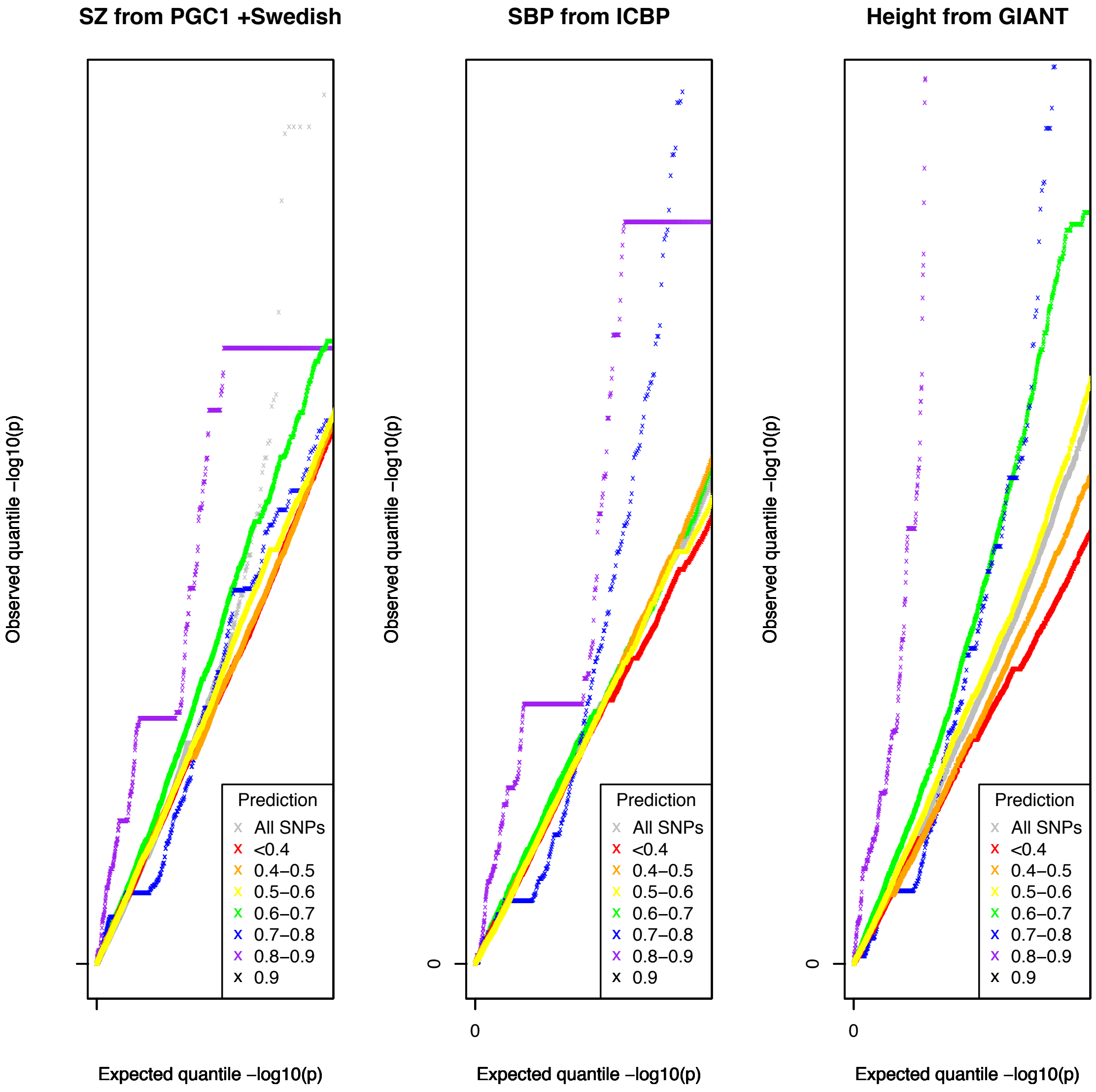

Figure S2. Quantile-quantile plots stratified by predicted values for SNPs in real GWAS. Only SNPs from the test set were plotted. All GWAS SNPs (in grey) for a schizophrenia GWAS from PGC1 with a Swedish sample [**a**], a systolic blood pressure GWAS from ICBP [**b**], and a height GWAS from GIANT [**c**]. The non-grey lines show plots for SNPs binned according to their predicted value from the non-phenotype specific model. These plots are similar to the plot where SNPs from both the training and test sets were plotted, **Figure 7**.

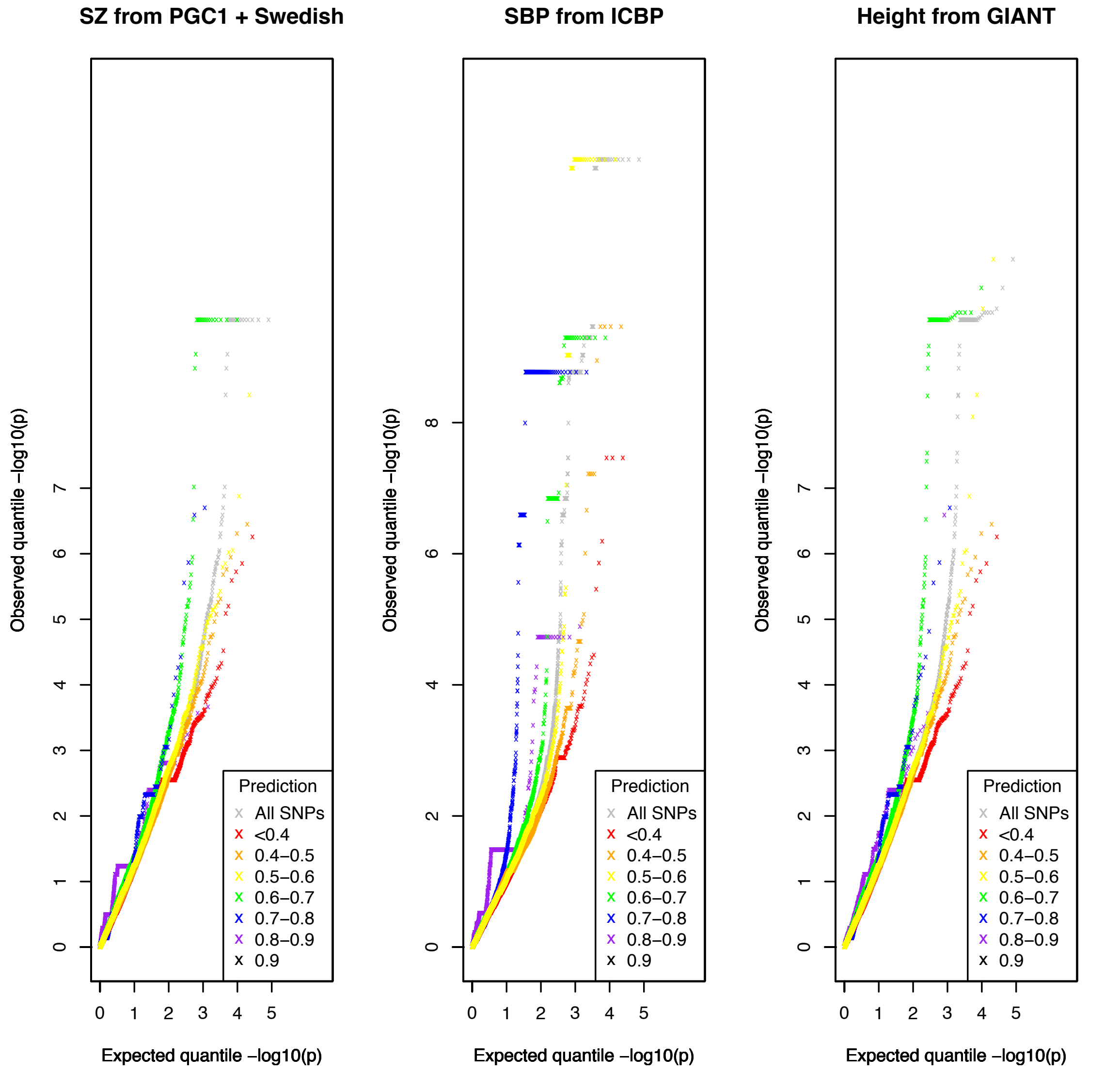

Figure S3. Quantile-quantile plots stratified by predicted values for SNPs in real GWAS. For each GWAS, prediction values were derived from models in which the genome-wide significant (p<5x10-8) hits were removed from the training set. All GWAS SNPs (in grey) for a schizophrenia GWAS from PGC1 with a Swedish sample [**a**], a systolic blood pressure GWAS from ICBP [**b**], and a height GWAS from GIANT [**c**]. The non-grey lines show plots for SNPs binned according to their predicted value from the non-phenotype specific model. These plots are similar to **Figure 7**, where the significant SNPs were not excluded from the training set for each respective GWAS.

Part D: Splitting of the data into training and test sets

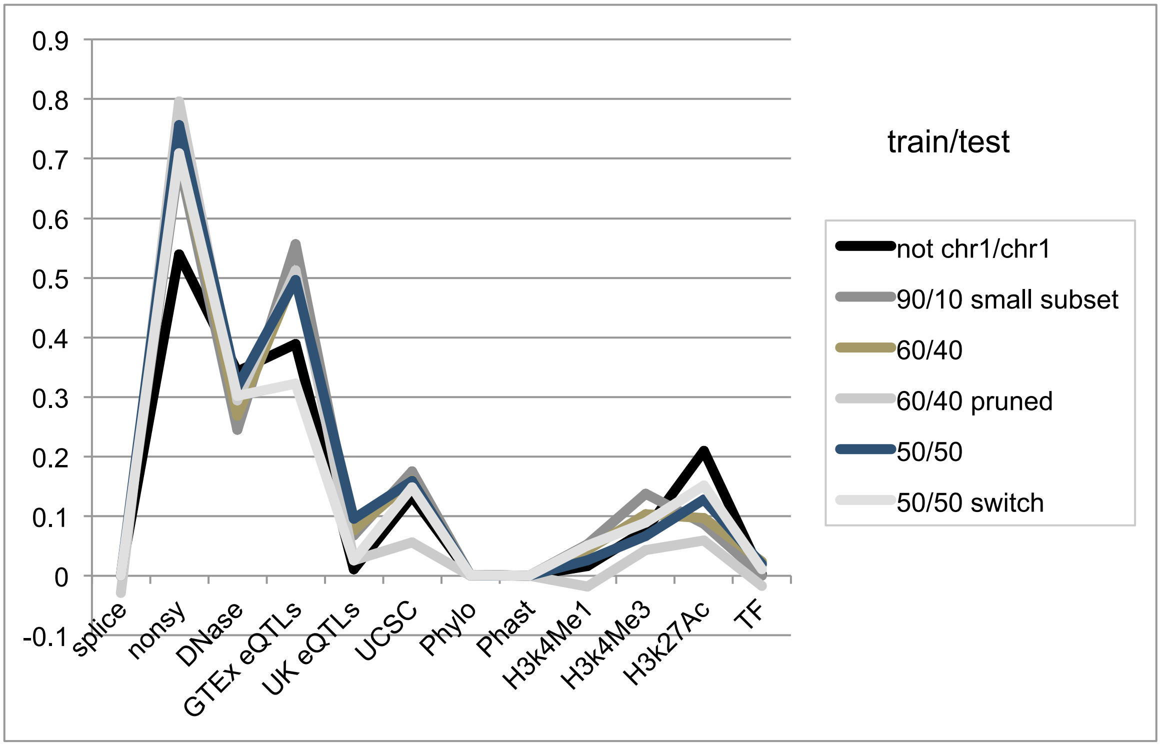

Figure S4. **Coefficients for functional characteristics in the clumped analysis for different training and test set proportions.** Comparison of beta coefficients that resulted from machine learning in the clumped non-phenotype specific analysis for various classifications of the training and test sets.

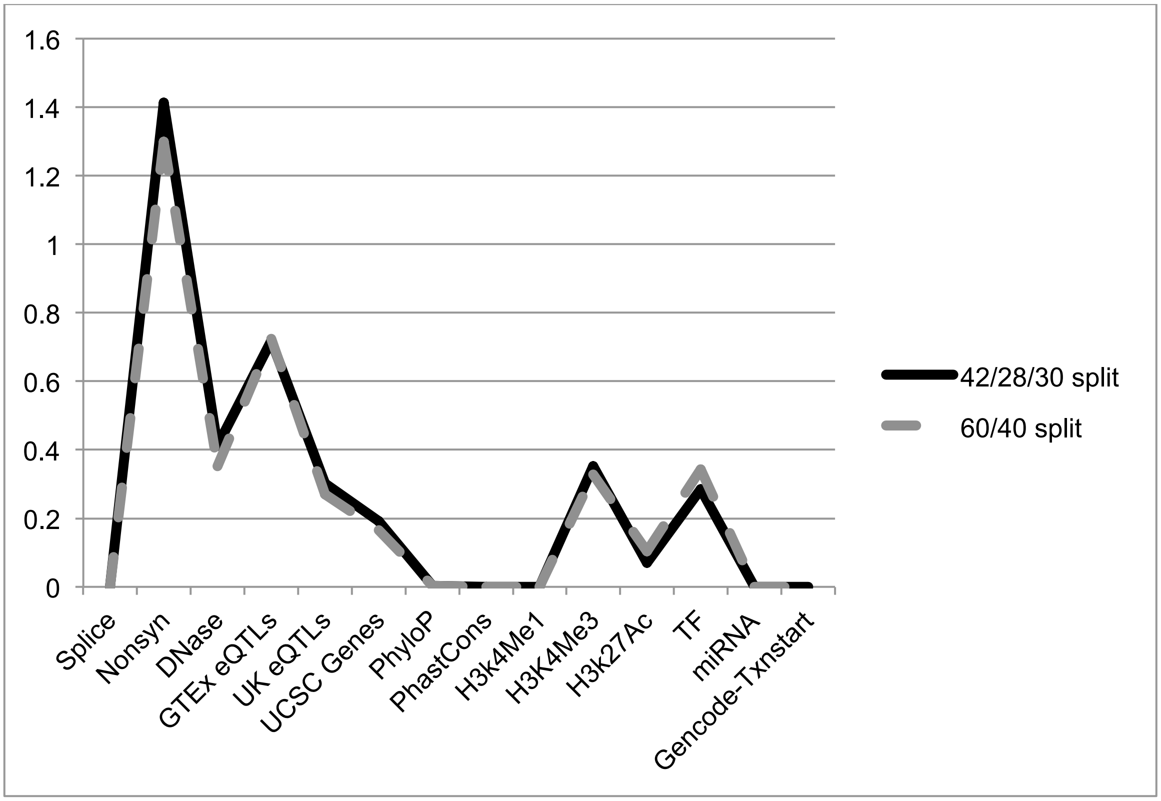

Figure S5. **Coefficients for functional characteristics in the clumped analysis when trained the model and tuned the parameters on independent sets.** Comparison of beta coefficients that resulted from machine learning in the clumped non-phenotype specific analysis when using a 42%/28%/30% split for training the model, tuning the parameters, and testing the model, respectively. (The 42% and 28% refer to 60% and 40% of 70%, respectively.) This model was compared to using a 60%/40% split where the training and tuning were conducted on the same set.

Part E: Variability of Beta values

Comparing the differences between beta values for the different seeds in the clumped analysis

There were some inconsistencies, interestingly, with the functional characteristics that have the lowest frequencies: for instance, for splice sites with the autoimmune analysis (**Table C**).

Table C. Beta values for "splice sites" for autoimmune clumped analysis.

seed1	seed2	seed3
0.18	0	0.34

Findings when looking at the distribution of annotated SNPs in the training sets for the different seeds:

For "splice sites" in the autoimmune analysis, seed2 only had one splice site that was also a GWAS hit in the training set.

Thus, betas are not always reliable for the low frequency characteristics. One needs to double check that the distribution of annotated SNPs in the GWAS hits in the training set is consistent. This conclusion is a caveat for the separated analysis since the frequencies for many of the annotated SNPs are very small.

Further investigation into the inconsistencies in the beta coefficients in the clumped analysis (all SNPs in the GWAS Catalogue)

To further investigate the hypothesis that beta coefficients are more variable among the different assignments of SNPs to the training and test sets for the low frequency characteristics, we assessed the relationship between the variance of betas and the frequency of the characteristic in the GWAS hits for the clumped non-phenotype specific analysis. In order to assess the variance we performed machine learning using all of the GWAS hits using 10 different seeds. Generally, the lower the frequency in the hits, the larger the variability of the beta coefficients for that particular functional characteristic (**Figure S6**).

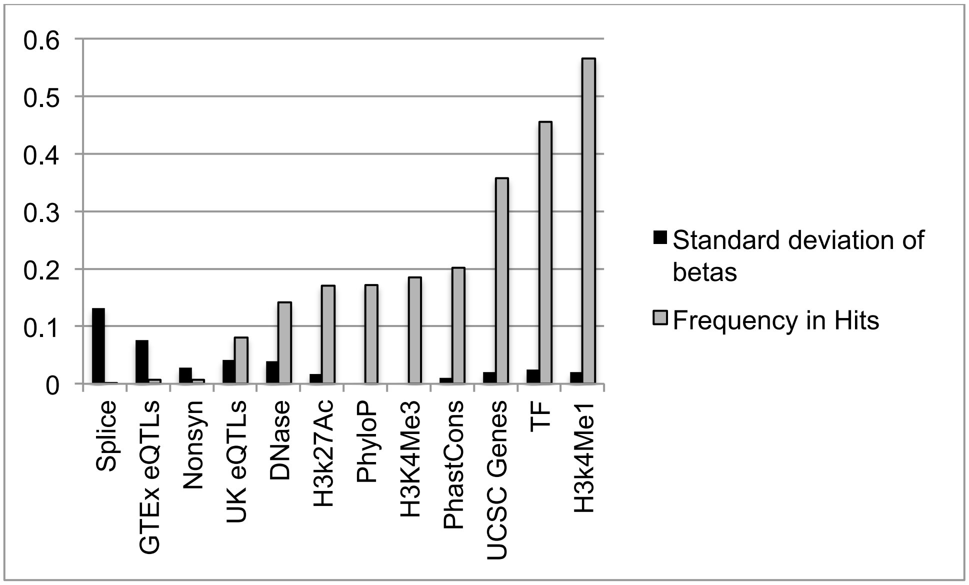

Figure S6. Standard deviation and frequency of functional characteristics. Relationship between the standard deviation of the beta coefficients derived from the machine learning performed 10 times using 10 different seeds in the random number generator that distributes the SNPs into the training and test sets, and the frequency of the functional characteristics in the GWAS hits.

Next, we investigated whether the betas would be stabilized among the different seeds if all functional characteristics were forced to be included in the model, which can be achieved through ridge regression. Ridge regression was performed for 10 different seeds, but the variability of the betas seen when using elastic net persisted (**Figure S7**).

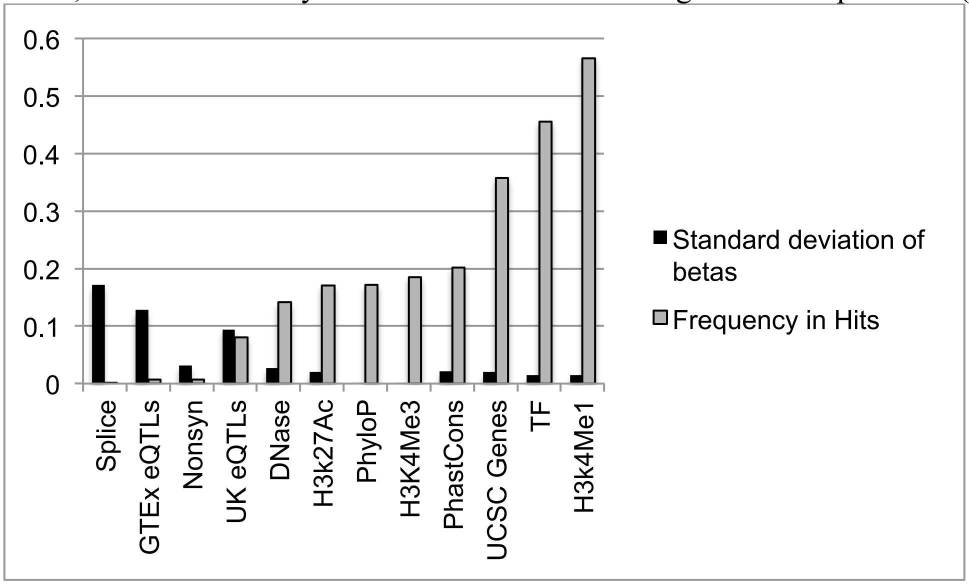

Figure S7. Standard deviation from ridge regression and frequency of functional characteristics. Relationship between the standard deviation of the beta coefficients derived from the ridge regression performed 10 times using 10 different seeds in the random number generator that distributes the SNPs into the training and test sets, and the frequency of the functional characteristics in the GWAS hits.

Comparing the differences between beta coefficients for the different seeds in the separated non-phenotype specific analysis (all SNPs in the GWAS Catalogue)

The frequencies of the functional characteristics in the separated analysis were quite low, and so we investigated whether the variability of the beta coefficients observed in the clumped analysis was also exhibited in the separated. We plotted the differences between the betas for the different seeds (**Figure S8**). The betas seem to be fairly consistent among the different seeds except for the transcription factor XRCC4 (the position of the largest peaks in the plot). In light of the results from the clumped analysis in which the low frequency functional characteristics presented inconsistencies, we looked at the characteristics with the lowest frequencies in the separated analysis (**Table D**), and XRCC4 was one of them. XRCC4 had variable betas: 0.3 for seed 1, zero for seed 2, and 0.46 for seed 3. However, the other two of the low frequency functional characteristics, EBF1C-8 and Pol2-4H8, had betas of zero for all three seeds.

Table D. **Lowest frequency functional characteristics in the non-phenotype specific separated analysis.**

Functional characteristic	Frequency in hits	Frequency in non-hits
EBF1C-8	0	2.49E-05
XRCC4	0.000118977	6.02E-05
Pol2-4H8	0.000118977	8.66E-05

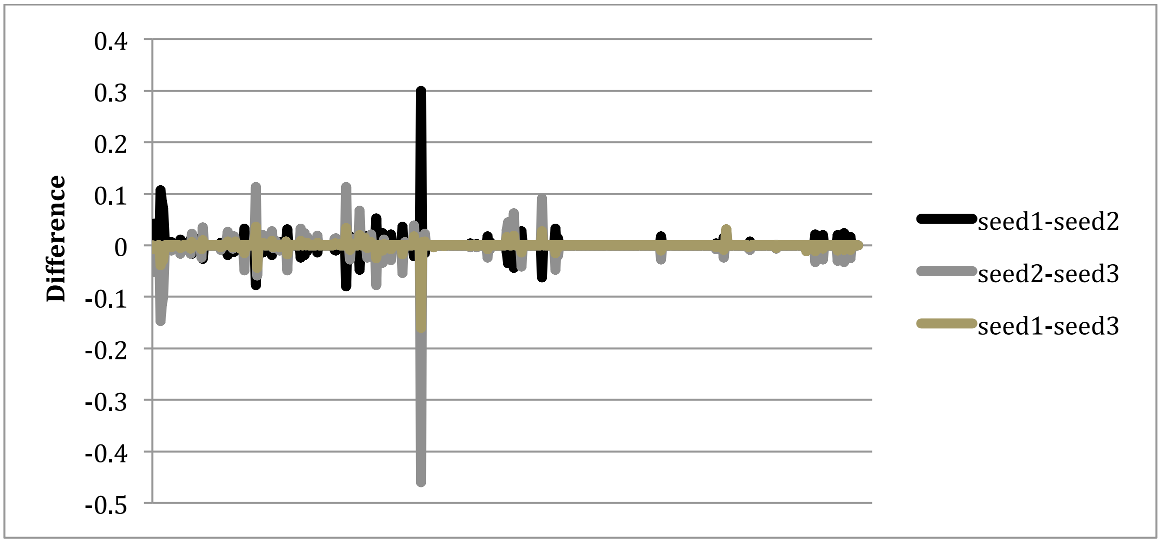

Figure S8. **Coefficients for the various functional characteristics in the separated analysis.** Plot showing the differences of between beta coefficients for different random number generators (seeds) for the functional characteristics in the separated non-phenotype specific analysis using all SNPs in the GWAS Catalogue.

Part F- Phenotype specific analysis using EFO defined categories

Due to the small size of the lists (not including "other disease" or "other measurement", which both lack biological relevance), it is not feasible to use the EFO classifications. **Table E** shows the number of GWAS hits that fall into each category. The numbers provided in the table are inflated as they assume that all of those SNPs are present on the GWAS arrays analysed in our analysis and that none of them are in the MHC region (which was excluded for the machine learning). Thus, the lists for training and testing are around 100 SNPs less than the listed values.

There were no results for the GWAS list for "biological processes" (ie. the betas were all zero), so machine learning on other lists with a smaller number of SNPs was not performed. Machine learning was also not run on the lists that lacked biological relevance even if they were larger than the list for "biological processes": for example: "other disease", "other measurement", and "other trait".

Table E. **EFO phenotype specific GWAS lists.**

**Phenotype**	**N in GWAS Catalogue (Aug. 6, 2013)**
Biological process	616
Metabolic disease	389
Mental disease	827
Immune disease	349
Hematological Measurement	284
Digestive disease	468
Cardiovascular disease	356
Cancer	685
Body measurement	639
Nervous system	680
Other Disease	1231
Other measurement	3216
Other trait	211
Drug response	593

Performing machine learning on the "biological process" list produced betas of zero (for at least two seeds), and thus was uninformative. The results from machine learning performed on the cancer list seemed more fruitful, producing an area under a ROC curve of 0.6. However, the magnitude of the betas and Bayes factors were much lower than those from the overall analysis. For instance, the largest Bayes factor when training on the cancer hits was 1.98, which lacks usefulness. We also tried to combine some of the "similar" lists together to create larger lists on which to train. For instance, the following combined lists were created: "mental/behavioural" + "nervous system" (N=1282), and "digestive disease" + "metabolic disease" (N=759). The list for "cardiovascular disease" was expanded by adding the SNPs for blood pressure, which is measured in the context of hypertension from "other measurement", but the overall list was too small for analysis (N=350). However, the use of combined lists distorts the motivation of using more reproducible phenotype specific lists, which was the rationale for looking at using the EFO definitions.
